# Exploration of the Reasons for Poor Adherence Among Metabolic Syndrome Patients Attending a Tertiary Care Hospital in Tamil Nadu, India: A Mixed Method Study

**DOI:** 10.7759/cureus.74753

**Published:** 2024-11-29

**Authors:** Megan Sarah Mathew, Vijayakarthikeyan M, Kavinkumar Saravanan, Angeline Grace, Angayarkanni P

**Affiliations:** 1 Undergraduate, Vinayaka Mission’s Kirupananda Variyar Medical College and Hospitals, Vinayaka Missions Research Foundation (Deemed to be University), Salem, Tamilnadu, IND; 2 Community Medicine, Vinayaka Mission’s Kirupananda Variyar Medical College and Hospitals, Vinayaka Missions Research Foundation (Deemed to be University), Salem, Tamilnadu, IND; 3 Community Medicine, Swamy Vivekanandha Medical College Hospital and Research Institute, Thiruchengode, Namakkal, The Tamilnadu Dr. M.G.R. Medical University, Namakkal, Tamilnadu, IND; 4 Community Medicine, Sree Balaji Medical College and Hospital, Bharath Institute of Higher Education and Research (BIHER) Deemed to be University, Chennai, Tamilnadu, IND; 5 Community Medicine, Sri Devaraj Urs Medical College, Sri Devaraj Academy of Higher Education and Research, Kolar, Karnataka, IND

**Keywords:** compliance, diet, lifestyle disease, metabolic syndrome (ms), mixed method research

## Abstract

Background

A major challenge in the treatment of MetS is the prevalence of low rates of adherence to the treatment regimen for individual components by the affected persons. This study aimed to estimate the medication adherence level among those with metabolic syndrome, determine the factors significantly associated with low adherence to medication, and explore the reasons for poor adherence to medication

Materials and methods

This sequential explanatory type of mixed method study was conducted among the metabolic syndrome patients attending the lifestyle clinic of a tertiary care hospital in the Salem district of Tamil Nadu, India. For the quantitative component, 210 was the sample size and for the qualitative component, the sample size was six. Adherence to drugs was assessed using the Morisky Medication Adherence eight-item Scale (MMAS 8).

Results

The mean age of the participants was 56.6+11.5 years with a female preponderance of 108 (51.5%). Medication adherence was low in 90 (42.7%) participants. A high degree of adherence was noted only in 41 (19.4%) participants. Multilogistic regression analysis revealed that patients on both oral drugs and insulin, patients with higher perceived stress levels, and those on low levels of physical activity were significantly associated with poor adherence.

Conclusion

Regular counseling and reinforcement by the treating physician to improve adherence to medication at the grassroots level is important. Information, Education, and Communication and Behaviour Change Communication methods can be adopted to improve knowledge and to bring about a change in behavior in order to curb this problem.

## Introduction

Metabolic syndrome (MetS) is caused by the interplay of various lifestyle and genetic factors namely ethnicity, age >40 years, cigarette smoking, alcohol consumption, sedentary lifestyle, overweight, obesity, and positive family history of type 2 diabetes mellitus (T2DM) [1,2]. Individuals with MetS have a three to five-fold increased risk of developing T2DM [3]. Globally, the prevalence of MetS varies between 7.9-43% in males and 7-56% in females, respectively [4].

In Southeast Asian countries, in addition to the rising prevalence of MetS, the age of onset of MetS is declining and this is due to increased consumption of high-calorie diets in younger age groups and also due to genetic predisposition [5]. Because of this scenario, Southeast Asian regions face serious health-related repercussions as they constitute 20% of the global population, and healthcare infrastructure in the majority of the nations is not sufficient to deal with this burden [6].

A major challenge in the treatment of MetS is the prevalence of low rates of adherence to the treatment regimen for individual components by the affected persons [7]. Monitoring of the individual components of the MetS also poses difficulties to the practitioners. Factors such as polypharmacy, economic constraints, and misconceptions about medication often deter patients from complying with prescribed regimens. Furthermore, inadequate patient-provider communication and insufficient follow-up care exacerbate non-adherence. To address these gaps, this study seeks to assess the levels of medication adherence among individuals with MetS and explore the barriers to adherence through a mixed-method approach.

Studies in resource-constrained settings have highlighted unique challenges to adherence, including poverty, lack of education, high treatment costs, cultural beliefs, and insufficient patient-provider communication. In South Asia, additional barriers include stigma, gender disparities in healthcare access, and high rates of illiteracy, all of which contribute to poor adherence. For example, in India, adherence rates among MetS patients are reported to be as low as 16.6% in some populations, reflecting a dire need for targeted interventions​ [8].

Medication adherence is defined as the degree to which a patient follows the prescribed dietary, lifestyle, and pharmacological recommendations from their healthcare provider. Adherence is particularly crucial for managing chronic diseases like MetS, as lifelong treatment regimens are often required to prevent complications. However, adherence rates for chronic conditions remain suboptimal, influenced by various factors such as socioeconomic status, literacy levels, awareness about the disease, and the complexity of treatment regimens. Low adherence rates can lead to poor glycemic control, unregulated blood pressure, and worsening lipid profiles, ultimately increasing the risk of life-threatening complications. Hence, it implies the active and voluntary commitment of the patient to produce a therapeutically beneficial result. The patient may be consuming multiple forms of medication to treat several other conditions. This creates a difficult situation for the patient where the patient has to be on multi-drug therapy and in these individuals, adherence plays a key role in achieving the desired outcome [9].

Despite its significance, data on adherence among MetS patients in the Indian context remains limited, particularly regarding the determinants of poor adherence. Most existing studies focus on adherence to single disease conditions like diabetes or hypertension, neglecting the multidimensional challenges faced by individuals managing MetS. This gap underscores the need for a focused investigation into adherence barriers, especially in settings where healthcare resources are constrained.

Adherence gains its utmost importance in chronic diseases as the treatment is usually given lifelong and the incidence of life-threatening complications can be avoided by strict adherence to management protocols. Adherence is affected by socioeconomic status, literacy, memory of the patient, lack of proper knowledge and awareness, duration of therapy, and certain other factors. Available literature indicates that adherence levels among individuals with MetS are scarce [10]. In order to overcome the information gap, this mixed method study was planned to estimate the adherence levels among those with MetS. Also, the qualitative component was analyzed to give the reasons for poor adherence. This study will shed some light on the existing problem.

## Materials and methods

A sequential explanatory type of mixed-method study design was employed [11]. The quantitative component was followed by the qualitative research method. This particular type was chosen, as the study's quantitative method findings were explained by the in-depth interviews administered to the purposively selected study participants. The study was performed in the Lifestyle Clinic of a Tertiary Care Hospital, Vinayaka Mission’s Kirupananda Variyar Medical College and Hospitals, Salem, Tamil Nadu, India. The study was conducted for a duration of six months from March 2023 to August 2023. The study was started after receiving approval from the Institutional Ethics Committee of Vinayaka Mission’s Kirupananda Variyar Medical College (approval number: VMKVM/IEC/23/001). Informed consent was obtained from each participant prior to the interviews. The Informed Consent form was prepared in both English and Tamil languages according to Indian Council of Medical Research (ICMR) guidelines. 

Sample size calculation

The sample size for the study was calculated to be 210 for the quantitative component using the formula (Zά)²pq/d^2^, where p is the prevalence of MetS among diabetes patients, taken as 49.5% based on a study conducted by Behera et al. in Odisha in 2018 [12]. Using a relative precision of 7.4% and 15% for non-response, the final sample size was rounded off to 210.

Inclusion and exclusion criteria

Inclusion Criteria 

The study included adults aged 18 years and above diagnosed with MetS according to the National Cholesterol Education Program Adult Treatment Panel III (NCEP ATP III) criteria. Participants were required to have been on prescribed medications for MetS, such as antihypertensives, lipid-lowering agents, or antidiabetic drugs, for a minimum duration of six months. Only those who provided informed consent were included in the study. Additionally, the participants were selected from patients attending outpatient or inpatient services at the tertiary care hospital during the study period.

Exclusion Criteria

Patients with cognitive impairments or mental health conditions that hinder accurate reporting of medication adherence were excluded. Individuals with acute illnesses or requiring hospitalization during the study period were also excluded, as such conditions could temporarily affect adherence patterns. Pregnant and lactating women were not included due to differences in metabolic profiles and medication adherence behaviors. Furthermore, patients with other chronic diseases requiring complex treatment regimens, such as malignancies or chronic renal failure, were excluded to avoid confounding factors. Lastly, participants unable or unwilling to provide informed consent were not considered for the study.

For the qualitative component, six MetS patients from the poorly compliant group were selected for in-depth interviews till the point of achievement of saturation of information was provided by the participant. For the quantitative part, patients were selected randomly based on the simple random sampling method using computer-generated random number tables. For in-depth interviews, a homogenous type of purposive sampling method was used. The participants were homogenous as they all belonged to the poor adherence group.

This study was performed in two phases. Phase 1 was done to estimate the sociodemographic details and adherence to treatment among MetS patients using a cross-sectional analytical study. Socioeconomic status was assessed by using a modified BG Prasad scale for 2024 [13]. The next phase of exploring the reasons for poor adherence to treatment was done using a qualitative study. All the diabetic patients visiting the clinic were screened for MetS using the NACP ATP-III criteria. They were assessed for their adherence to drugs prescribed to treat their disease. The adherence to drugs was assessed using the Morisky Medication Adherence eight-item Scale (MMAS 8).

Operational definitions

MetS was defined based on the NCEP ATP III criteria. According to this, an individual is considered to have MetS if they meet three or more of the following conditions: central obesity with a waist circumference >102 cm for males or >88 cm for females, hypertriglyceridemia with triglyceride levels ≥150 mg/dL or specific medication use, low high-density lipoprotein (HDL) cholesterol (<40 mg/dL in males or <50 mg/dL in females, or specific medication use), hypertension (systolic blood pressure ≥130 mmHg or diastolic blood pressure ≥85 mmHg, or on antihypertensive medication), and fasting plasma glucose levels ≥100 mg/dL or a prior diagnosis of T2DM. These criteria highlight the systemic and multifactorial nature of MetS, underscoring the need for comprehensive management strategies [14].

Medication adherence was assessed using the MMAS-8, an eight-item questionnaire designed to evaluate the extent to which patients adhere to their prescribed treatment regimens. Each question carries a maximum score of 1, with a cumulative score classifying adherence levels as low (<6), medium (6-<8), or high(8). This tool captures various dimensions of adherence, such as forgetfulness, intentional skipping of medication, and challenges due to perceived inefficacy or side effects [15]. These operational definitions provided a standardized framework for categorizing the severity of MetS and evaluating adherence levels, enabling a systematic exploration of the factors contributing to non-adherence in this population.

An in-depth interview (IDI) was conducted among the purposively selected participants. This was conducted by the principal investigator (MSM) after proper training in qualitative research methods (QRM) and was guided by the co-investigator (VM) who was already trained in QRM. IDI was facilitated using a semi-structured interview guide to capture the reasons for poor adherence as perceived by the participants. By using IDI, a good rapport was developed between the participant and investigator, and this in turn resulted in achieving more trustworthy and highly reliable responses from study samples. Interviews were performed in the local vernacular language (Tamil) of the study participants. Each interview lasted about 30-40 minutes in a place convenient and comfortable to the participants. After obtaining their consent, the interviews were audio-recorded for doing content analysis.

A structured questionnaire was used as a study tool for data collection, by interviewing the study participants (see Appendices).

Statistical analysis

Data was entered in Epi Data Version 4.2 (EpiData Association, Odense, Denmark) and analyzed using IBM SPSS Statistics for Windows, Version 24.0 (Released 2016; IBM Corp., Armonk, New York, United States). Description analysis was summarized as mean with SD or frequency with proportion and analytical statistics were used. The association between risk factors and poor adherence was tested using the Chi-square test at 95% confidence interval (CI). Logistic regression was also done to identify the adjusted risk factors. A P-value of <0.05 was considered statistically significant.

Qualitative information was analyzed by manual content analysis. The audio taped interviews were transcribed by the Principal Investigator who was trained in carrying out qualitative data analysis. This enabled the grouping of similar responses across study participants and assisted in categorizing the findings and attaching them to the areas being explored. The guidelines by the University of California, Los Angeles (UCLA) Centre for Health Policy Research [16] were used for analysis. The Consolidated Criteria for Reporting Qualitative Research (COREQ) guidelines [17] were followed while reporting this qualitative work.

## Results

The mean age of the participants was 56.6 + 11.5 years, with a female preponderance (n=103, 51.5%). Nearly 165 (78.7%) patients were currently married, 79 (37.3%) had an education of higher secondary and above, and 22 (10.7%) were without any formal education. On the occupational front, 89 (42.2%) were homemakers and 73 (35.0%) were currently employed. Around 90 (43%) belonged to either upper middle or lower middle socioeconomic status as per the modified BG Prasad Scale for 2024 and the majority of patients (n=175, 83.5%) were from rural areas. Current smokers and current alcohol consumers were 4.9% and 8.3%, respectively (Table 1).

**Table 1:** Sociodemographic distribution of the study participants (N=210) * Modified BG Prasad scale 2024 Data is given as frequency and percentage, except for age, which is given as mean±SD

Characteristics	Frequency	Percentage
Age (years), mean ± SD	56.6±11.5	
Gender		
Male	102	48.5
Female	108	51.5
Marital Status		
Married	165	78.7
Single	4	1.9
Divorced/widowed	41	19.4
Education category		
No formal education	22	10.7
Primary	59	28.2
Secondary	50	23.8
Higher secondary	33	15.5
Graduate and above	46	21.8
Occupation		
Unemployed	5	2.4
Employed	73	35.0
Homemaker	89	42.2
Retired	43	20.4
Socioeconomic Status*		
Upper class	34	16
Upper middle/Lower middle	90	43
Upper lower	65	31
Lower	21	10
Type of Residence		
Rural	175	83.5
Urban	35	16.5

**Figure 1:**
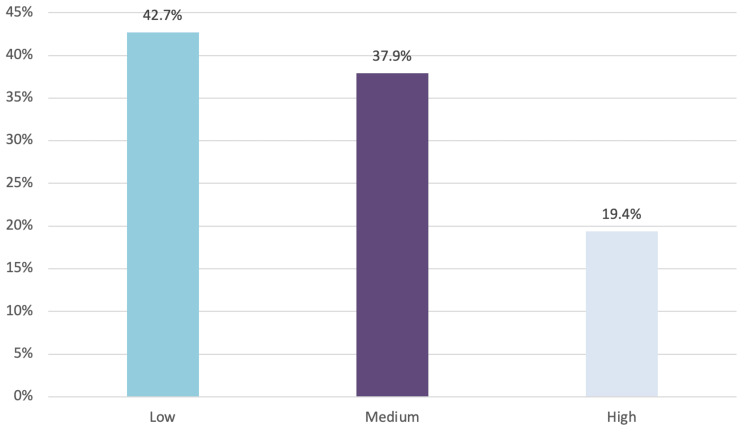
Proportion of medication adherence among the study participants (N=210)

Figure 1 illustrates the distribution of participants' adherence to medication as assessed by the MMAS-8. A substantial proportion of participants (n=90, 42.7%) demonstrated low adherence (scores <6), indicating significant challenges in consistently following prescribed treatment regimens. Moderate adherence (scores 6-<8) was observed in 79 (37.9%) participants, while only 41 (19.4%) achieved high adherence (score of 8), highlighting the limited number of patients fully complying with their medication.

Table 2 presents the multivariate logistic regression analysis showing associations between various covariates and low medication adherence among the study participants. Patients on a combined treatment of oral hypoglycemic drugs and insulin were significantly more likely to exhibit low adherence (Adjusted prevalence ratio (PR) = 1.35, 95%CI: 1.05-1.75, p = 0.021). Higher perceived stress levels were also associated with low adherence, with patients reporting high stress (27-40 on the scale) having a significantly increased likelihood of poor adherence (Adjusted PR = 1.31, 95%CI: 1.04-1.65, p = 0.021). Similarly, participants with low levels of physical activity (0-599 Metabolic Equivalent of Tasks (METs)) were at a higher risk of low adherence compared to those with moderate activity levels (Adjusted PR = 1.37, 95%CI: 1.06-1.76, p = 0.015). Gender, insulin-only treatment, and moderate stress levels did not show significant associations with low adherence, highlighting specific modifiable factors such as stress and physical activity in improving adherence rates.

**Table 2:** Multivariate logistic regression showing the association between the co-variables and low adherence to medication (N=210) *p-value statistically significant (<0.05) METS: Metabolic Equivalent of Tasks; PR: prevalence ratio

Co-variables	Unadjusted PR (95% CI)	p value	Adjusted PR (95% CI)	p value
Gender				
Male	Reference		Reference	
Female	1.24 (0.97-1.59)	0.080	1.19 (0.79-1.79)	0.396
Type of treatment				
Oral hypoglycemic drugs	Reference		Reference	
Oral hypoglycemic drugs + Insulin	1.35 (1.04-1.74)	0.020^*^	1.35 (1.05-1.75)	0.021*
Insulin	1.09 (0.73- 1.65)	0.651	1.05 (0.69-1.59)	0.819
Perceived stress level				
Low (0-13)	Reference		Reference	
Moderate (14-26)	1.17 (0.89- 1.54)	0.266	0.99 (0.77-1.29)	0.986
High (27-40)	1.45 (1.09-1.93)	0.009^*^	1.31 (1.04-1.65)	0.021^*^
Level of physical activity				
Low (0-599 METs)	1.36 (1.04-1.79)	0.026^*^	1.37 (1.06-1.76)	0.015^*^
Moderate (600-2999 METs)	Reference		Reference	
High (≥3000 METs)	NA	0.991	NA	0.993

Qualitative section

**Figure 2:**
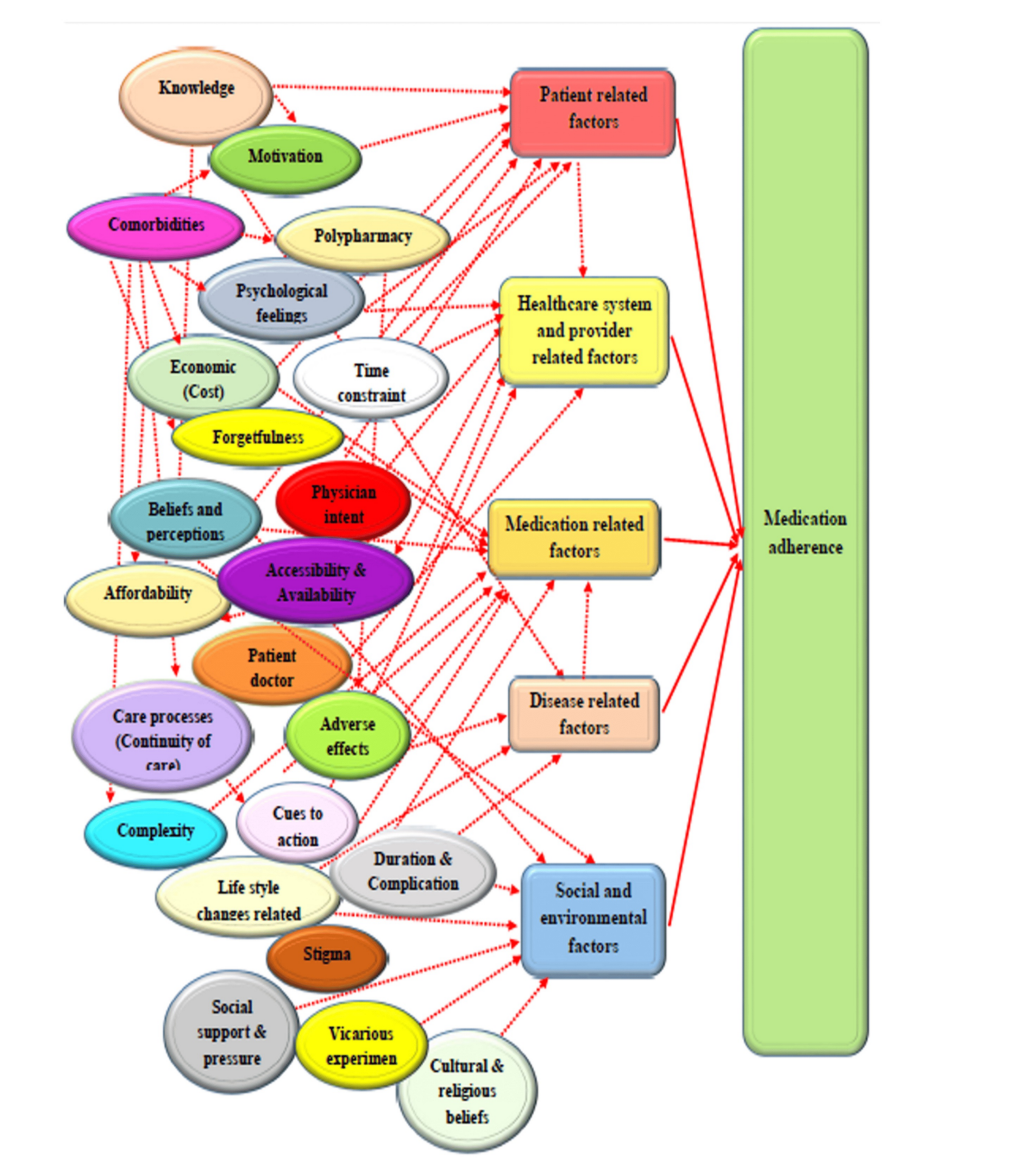
Conceptual framework (Barriers to Medication Adherence) Image Credit: Authors

For the qualitative interview, six IDIs were conducted among the patients with low and medium level of adherence. Three participants from the medium level of adherence and three participants from the low level of adherence were included. Grounded theory approach was used to arrive at the themes and subthemes given in Figure 2.

Theme 1: Patient-Related Factors

Patient-related barriers such as lack of knowledge, misbeliefs, and forgetfulness were significant contributors to poor adherence. Many participants lacked awareness of advanced treatments like insulin pens: "I have heard about them but don’t know much about their use" (51-year-old female patient on insulin). Misbeliefs about disease control were common, as one participant stated, "I stopped eating sugars and assumed I didn’t need regular medications" (37-year-old female patient). Forgetfulness due to workload was another issue: "I’m too occupied with household work to remember my medications" (51-year-old female patient).

Theme 2: Healthcare System and Provider Factors

Barriers related to healthcare accessibility and processes included affordability, distance, and limited interaction with providers. One participant shared, "Insulin is not available nearby, and I have to travel far to get it" (51-year-old female patient on insulin). Others felt disconnected during doctor visits: "The doctor just writes the medicine and sends me off" (31-year-old male patient). These issues highlight gaps in continuity of care and provider engagement.

Theme 3: Medication-Related Factors

Polypharmacy, complexity of regimens, and adverse effects hindered adherence. Patients found managing multiple drugs overwhelming: "It’s too laborious taking multiple injections daily" (50-year-old female patient on oral drugs and insulin). Adverse effects like dizziness were also cited: "I feel dizzy after taking medications, so I skip them" (50-year-old female patient). Such challenges emphasize the need for simplified treatment plans.

Theme 4: Social and Environmental Factors

Cultural beliefs, social support, and stigma influenced adherence. One participant noted, "I skip my medications on fasting days" (50-year-old female patient on insulin). Another cited a lack of family support: "None of my family members live with me now" (75-year-old male patient on oral drugs). Social factors and isolation compounded the difficulty of maintaining adherence.

Theme 5: Disease-Related Factors

The asymptomatic nature of MetS and perceived disease control led to non-adherence. One participant stated, "I feel healthy and don’t have symptoms, so I thought I didn’t need medications" (37-year-old female patient). This highlights the critical role of patient education in addressing misconceptions about disease management.

## Discussion

Our study reported that medication adherence was low in 42.7% of the participants. The high degree of adherence was noted only in 19.4% of the participants. The studies conducted across various parts of the globe support this finding, where high adherence was seen in the studies conducted by Arifulla et al. [18] in UAE and Broadbent et al. [19] in New Zealand where the adherence to medication was quite high: 84% and 86%, respectively. This difference underscores the influence of robust healthcare infrastructure, better patient education, and easier access to medications in developed nations. Patients in these settings likely benefit from supportive healthcare systems with regular follow-ups, personalized care, and financial resources that promote adherence.

However, the studies conducted in India showed a lower adherence to medication ranging from 16.6% to 51.6% [20-22]. This is further supported by the study conducted by Amende et al. where the non-adherence rate was similar to our study (45.6%) [23]. Hence, it could be well argued that developing countries are still facing high levels of non-adherence. These barriers include limited healthcare access, higher costs of medications, and the logistical challenges of rural populations seeking care in urban facilities. Additionally, cultural beliefs, lack of awareness, and lower health literacy further complicate adherence in such settings. This supports the assertion that developing nations face unique challenges in managing chronic conditions like MetS.

We developed a model to explore the reasons for non-adherence and it was found that patient-related factors, healthcare system and provider-related factors, medication-related factors, disease-related factors, and social and environmental factors were the main reasons behind the non-adherence to medication among our study population. It could be well noted that our study results almost correlated with various other quantitative study findings [18,20-23]. Forgetfulness, awareness about the disease, and polypharmacy along with the motivation to stick to the medication schedule were quoted as the main reasons for non-adherence by many participants. Other reasons such as social factors and lack of affordability combined with availability were also quoted by some participants for non-adherence.

The qualitative findings provided important insights into the factors influencing adherence, particularly concerning stress levels and physical activity, which were also significant in the quantitative analysis. Participants frequently cited work overload, economic instability, and lack of social support as key stressors, directly impacting their ability to adhere to treatment regimens. These findings align with the logistic regression results, underscoring the role of psychosocial stress in poor adherence. Similarly, the qualitative data revealed that low physical activity was influenced by barriers such as lack of awareness, insufficient motivation, and cultural norms prioritizing other daily responsibilities over exercise. Actionable interventions to address these challenges could include incorporating stress-reduction programs, such as mindfulness or counseling sessions, into routine care and community-based initiatives to promote physical activity, tailored to cultural and economic contexts [22].

Additionally, while socioeconomic status and rural-urban differences were considered, their potential confounding effects were not deeply explored. Future analyses should assess whether disparities in healthcare access or financial resources exacerbate adherence barriers. Finally, triangulating qualitative findings with quantitative outcomes reveals critical intersections, such as the impact of polypharmacy and cultural beliefs. Patients reported difficulty managing multiple medications and expressed misconceptions about disease seriousness, directly reflecting the quantitative association between complex treatments and poor adherence. Integrating educational interventions, leveraging community health workers, and streamlining treatment regimens could help address these intertwined barriers effectively.

One of the major strengths of the study is that it is the first of its kind to develop a model to explore the reasons behind non-adherence to medication. We employed a representative sample of the population and hence the study results could be generalized to the population of a similar setting. One limitation of the study is the subjective nature of the measurement tool for assessing non-adherence to medication which could have brought in the component of social desirability bias in our study. Self-reporting has the benefits of being easy to use, not being intrusive, and also providing information on the attitudes of patients and different beliefs about medication use. The limitations of self-reporting are difficulty in understanding, and participant’s willingness to disclose personal data. These can affect the response accuracy and the questionnaire validity. One other limitation is that our study is a cross-sectional study and the factor of reverse causation could not be ruled out, as with all other cross-sectional studies. The diversity of patient experiences may not be well represented by the qualitative sample (n=6), which is also a limitation. 

To address the identified barriers, several measures can be implemented. First, patient education programs should focus on dispelling myths about disease management and emphasizing the importance of adherence. Counseling sessions could include visual aids and interactive discussions to improve patient understanding. Second, healthcare systems need to enhance the accessibility and affordability of medications, especially for rural populations. Policies that support subsidized medications and streamline access to healthcare facilities can significantly improve adherence. Third, providers should adopt patient-centered approaches, including frequent follow-ups and personalized treatment plans to reduce the complexity of regimens.

Moreover, incorporating stress management interventions, such as mindfulness and relaxation techniques, could help patients cope with the psychological burden of chronic disease. Encouraging physical activity through structured programs or community-based initiatives may also improve adherence indirectly by promoting overall well-being. Finally, future research should explore innovative adherence-monitoring tools, such as digital health apps, to provide real-time feedback and reminders to patients.

## Conclusions

The study identifies significant barriers to medication adherence among patients with MetS, including high perceived stress, low physical activity, and complex treatment regimens. Addressing these modifiable factors requires a comprehensive approach. Patient-centered interventions such as stress management programs and simplified medication regimens should be coupled with systemic changes such as improving medication affordability and ensuring consistent provider engagement. Furthermore, targeted educational campaigns could address knowledge gaps and cultural beliefs that hinder adherence. Future research should explore the effectiveness of these interventions in similar low- and middle-income country contexts to inform broader policy and practice. Addressing these challenges through tailored interventions and systemic enhancements has the potential to improve adherence and lead to better long-term health outcomes for this population.

## Appendices

Questionnaire

**Table 3:** Questionnaire used in the study HDL: high-density lipoprotein; TGL: triglyceride level; FBS: fasting blood sugar; RBS: random blood sugar

Section	Questions/Parameters	Options
Demographics	Respondent ID Code	
	Age	Open-ended values
	Sex	Male/Female/Others
	Hospital Number	Open-ended values
	Address	Open-ended
	Marital Status	Married, Unmarried, Divorced, Widowed
Educational Status	Level of Education	No formal education, Primary, Secondary, Higher Secondary, Graduate and above
Occupation	Employment Status	Unemployed, Employed, Homemaker, Retired
Residency	Residing Area	Rural, Urban
	Distance from Hospital	<5 kms, 5–10 kms, >10 kms
Income	Monthly Income	₹10,000
Medication	Were you prescribed any medication for your health condition?	Yes, No
	How many types of medication were prescribed?	Oral tablets only, Injections only, Combined (both)
Medication Adherence	Do you sometimes forget to take your medicine?	Yes, No
	In the past 2 weeks, were there any days you missed your medication for reasons other than forgetting?	Yes, No
	Have you ever stopped taking medication without informing your doctor because it made you feel worse?	Yes, No
	Do you sometimes forget to bring your medication while traveling?	Yes, No
	Did you take all your medicines yesterday?	Yes, No
	When symptoms feel under control, do you stop taking your medication?	Yes, No
	Do you feel hassled about sticking to your treatment plan?	Yes, No
	How often do you find it difficult to remember taking all your medications?	Never/Rarely, Once in a while, Sometimes, Usually, All the time
Perceived Stress Scale	Series of 10 questions on stress in the past month (e.g., "How often have you felt nervous and stressed?")	Scored 0–4 for each question
Physical Activity Adherence	Days and duration of vigorous, moderate, or walking activities; time spent sitting	Open-ended responses (Days/Hours/Minutes)
Dietary Modification Adherence	Do you follow dietary advice given by your physician?	Daily, Frequently, Rarely, Never
	Reasons for not following dietary advice	Lack of discipline, Forgot details, No advice given, Doesn’t affect health
Lifestyle Modification Adherence	Smoking Status	Current smoker, Past smoker, Never smoked
	Alcohol Consumption	Current consumer, Past consumer, Never consumed
Follow-Up Adherence	Frequency of medical check-ups	As advised, Less than advised, More than advised
	Reasons for less frequent visits	Distance, Lack of transportation, Cost, Busy schedule, Feeling better, No accompaniment
Anthropometry	Waist circumference	Measured in centimeters
Laboratory Tests	HDL cholesterol, TGL levels, Blood pressure, FBS, RBS	Open-ended values

## References

[1] Ferrannini E (1995). Physiological and metabolic sequences of obesity. Metabolism.

[2] Grundy SM (2003). Inflammation, hypertension, and the metabolic syndrome. JAMA.

[3] Ford ES (2005). Risks for all-cause mortality, cardiovascular disease, and diabetes associated with the metabolic syndrome: a summary of the evidence. Diabetes Care.

[4] Balkau B (2004). Smoking, type 2 diabetes and metabolic syndrome. Diabetes Metab.

[5] Gupta R, Misra A, Vikram NK, Kondal D, Gupta SS, Agrawal A, Pandey RM (2009). Younger age of escalation of cardiovascular risk factors in Asian Indian subjects. BMC Cardiovasc Disord.

[6] Gupta M, Singh N, Verma S (2006). South Asians and cardiovascular risk: what clinicians should know. Circulation.

[7] DiMatteo MR, Giordani PJ, Lepper HS, Croghan TW (2002). Patient adherence and medical treatment outcomes: a meta-analysis. Med Care.

[8] (2003). Adherence to long-term therapies: evidence for action. Adherence to Long-Term Therapies: Evidence for Action.

[9] Kassahun A, Gashe F, Mulisa E, Rike WA (2016). Nonadherence and factors affecting adherence of diabetic patients to anti-diabetic medication in Assela General Hospital, Oromia Region, Ethiopia. J Pharm Bioallied Sci.

[10] Sontakke S, Jadhav M, Pimpalkhute S (2015). Evaluation of adherence to therapy in patients of type 2 diabetes mellitus. J Young Pharmacists.

[11] Hanson WE, Creswell JW, Clark VL (2005). Mixed methods research designs in counseling psychology. J Counsel Psychol.

[12] Behera BK, Khora PK, Pathi D (2018). A study on prevalence of metabolic syndrome and associated cardiovascular risk factors among diabetic patients attending a tertiary care hospital in eastern Odisha. J Evid Based Med Healthc.

[13] Javalkar SR, Davalagi S (2024). Socio economic status assessment in India: history and updates for 2024. Int J Comm Med Pub Health.

[14] Athyros VG, Ganotakis ES, Elisaf M, Mikhailidis DP (2005). The prevalence of the metabolic syndrome using the National Cholesterol Educational Program and International Diabetes Federation definitions. Curr Med Res Opin.

[15] Azharuddin M, Adil M, Sharma M, Gyawali B (2021). A systematic review and meta-analysis of non-adherence to anti-diabetic medication: evidence from low- and middle-income countries. Int J Clin Pract.

[16] (2024). UCLA Center for Health Policy Research: Key informant interviews. Section 4: Key Informant Interviews.

[17] Tong A, Sainsbury P, Craig J (2007). Consolidated criteria for reporting qualitative research (COREQ): a 32-item checklist for interviews and focus groups. Int J Qual Health Care.

[18] Arifulla M, Lisha Jenny JO, Sreedharan J (2014). Patients’ adherence to anti-diabetic medications in a hospital at Ajman, UAE. Malays J Med Sci.

[19] Broadbent E, Donkin L, Stroh JC (2011). Illness and treatment perceptions are associated with adherence to medications, diet, and exercise in diabetic patients. Diabetes Care.

[20] Mathew EM, Rajiah K (2013). Assessment of medication adherence in type-2 diabetes patients on poly pharmacy and the effect of patient counseling given to them in a multispecialty hospital. J Basic Clin Pharm.

[21] Premanandh K, Shankar R (2019). Adherence to treatment in patients with type 2 diabetes mellitus. Nat J Res Comm Med.

[22] Sharma T, Kalra J, Dhasmana D (2014). Poor adherence to treatment: a major challenge in diabetes. J Indian Acad Clin Med.

[23] Aminde LN, Tindong M, Ngwasiri CA, Aminde JA, Njim T, Fondong AA, Takah NF (2019). Adherence to antidiabetic medication and factors associated with non-adherence among patients with type-2 diabetes mellitus in two regional hospitals in Cameroon. BMC Endocr Disord.

